# Tropical Medicine

**Published:** 1918-09-07

**Authors:** 


### TROPICAL MEDICINE.

**London School of Tropical Medicine.**—Owing to war conditions the number of students attending the school has been greatly reduced, the ordinary curriculum has been suspended and short courses substituted These courses are principally attended by medical officers seconded for study by the War Office, and include officers of the R.A.M.C., I.M.S., and the Canadian and Colonial Forces—a certain number of other medical graduates are also in attendance. The war has robbed the school of the services of the Protozoologist, Bacteriologist, and all the demonstrators, but the honorary medical staff and the remaining teachers and lecturers are still carrying on the educational work of the establishment.

There is no diminution in the number of tropical cases admitted to the wards; indeed, there has been rather an increase, for officers suffering from tropical disease sent home from Mesopotamia, Egypt, and Eastern Europe are admitted for treatment under an arrangement with the War Office and India Office.

Though the reduced number of students has materially affected the financial position of the school, there have been many economies effected which have reduced the deficit to a minimum. The school offers to receive Belgian graduates and to afford them free tuition and board and residence.

Graduates desirous of attending for instruction in the laboratories or attending the cliniques in the wards of the Seamen's Hospital, Albert Dock, E., to which the school is attached, should apply to the acting Director at the school, or to the Dean, Sir Havelock Charles, at the India Office, Whitehall, S.W.

**Liverpool School of Tropical Medicine.**—The Liverpool School of Tropical Medicine is carried on in conjunction with the University of Liverpool and the Royal Infirmary, for the purposes of research and clinical teaching respectively. A special ward at the hospital has been set apart for the reception and treatment of tropical diseases, and facilities for investigation and study are given in the Johnston Laboratories of the University. The fee for a full course (lasting thirteen weeks) is 13 guineas. A short course, specially arranged for advanced instruction, is also held during June (fee 4 guineas).

**Both Cambridge University and the English Conjoint Board** hold examinations for the Diploma in Tropical Medicine. Neither of these bodies teaches Tropical Medicine; candidates derive their knowledge of the subject elsewheie, usually at either the London or the Liverpool School.

**Edinburgh Diploma of Tropical Medicine.** The University of Edinburgh grants a Diploma in Tropical Medicine and Hygiene. The examinations are held in December and June. The fees for the first and any subsequent appearance are: Practical bacteriology, £1 1s.; diseases of tropical climates, £1 1s.; tropical hygiene, £1 1s.; tropical clinical medicine, £1 1s.; total, £4 4s.

